# Editorial: AI processing of UAV acquired images for pattern monitoring in natural and urban environments

**DOI:** 10.3389/frobt.2022.1053063

**Published:** 2022-11-24

**Authors:** Yago Diez Donoso, Nuno Gracias, Mariano Cabezas, Carsten Juergens, Maximo Larry Lopez Caceres

**Affiliations:** ^1^ Faculty of Science, Yamagata University, Yamagata, Japan; ^2^ Underwater Robotics Research Center, Computer Vision and Robotics Institute, University of Girona, Girona, Spain; ^3^ Brain and Mind Centre, University of Sydney, Sydney, NSW, Australia; ^4^ Geomatics Group, Geography Department, Ruhr-University, Bochum, Germany; ^5^ Faculty of Agriculture, Yamagata University, Tsuruoka, Japan

**Keywords:** editorial, deep learning, UAV imaging, natural environments, urban environments, SLAM

Remote sensing Data, and especially data collected by Unmanned Air Vehicles (UAVs), is currently being used in an increasing number of research areas. These data, together with the new image analysis possibilities that Deep Learning provides, are pushing forward significant advances in research areas as diverse as Traffic Surveillance Bozcan and Kayacan (2020), Forestry (Diez et al., 2021), Agriculture Kamilaris and Prenafeta Boldu (2018) or Cultural Heritage inspection Themistocleous (2020). Our aim in this Research Topic is to explore how the combination of remote sensing data and AI (and very particularly Deep Learning) can be exploited to further widen their field of application, and to obtain better results in existing applications.

We believe that the four articles gathered in this Research Topic fulfill our goals and provide a very interesting perspective on how much this research area has already advanced in the last few years and how much further it can go. From novel takes on existing technology such as microdrones being used to obtain 3D-reconstructions of industrial buildings, to developments on the way data is collected (for example, semantic segmentation) to influence future data collection missions, and, finally, to results in established application areas, such as SLAM for agriculture or underwater natural environment monitoring, that transcend UAV data collection but use the same types of information and analysis approaches. We encourage the readers of this Research Topic to carefully examine the contributions presented but also to consider the future development possibilities that they open for these rapidly developing technologies.

The first contribution, Weißmann et al. presents a novel approach to construct 3D point clouds, with data from post-industrial buildings in the Rhine-Ruhr Metropolitan region in Germany. By using inexpensive micro-drones together with widely available open source software, the authors demonstrate how drones can act as new data sources that can change the way we visualize and relate to our environment. In particular, the authors show how the generated photorealistic 3D models can achieve accuracy similar to that of publicly available airborne laser scanning (ALS) data sets. Both the intelligent use of an inexpensive type of drone and the readiness of the technology convincingly emphasize the argument that this type of applications will continue to improve and enhance the perception of our environment. In conclusions, the authors express that their line of research should be further developed in other practical applications and single out the need to include cognitive feedback on the processing of data.

As an example of how research in fundamentally distinct practical applications in this field frequently uses similar techniques and creates interesting synergies, the second contribution Pimentel de Figueiredo et al. already serves as an answer to some of the questions that the authors of Weißmann et al. pose. Pimentel de Figueiredo et al. presents a new framework for autonomous mapping based on using geometric and semantic segmentation information computed with Deep Convolutional Neural Networks to improve the map representations used in Next-Best-View planning techniques. This paper represents a very interesting example on how to build on existing technologies, such as the off-the-shelf visual-inertial SLAM system used by the authors, and go beyond their capabilities by using the multi-sensor capacities of drones (in this case multiple cameras, Inertial Motion Units (IMUs), and an altimeter) and processing the data collected with state-of-the-art deep learning networks (in this case Bisenet Yu et al. (2018) for semantic segmentation).

Although the remaining two contributions, Silva Aguiar et al. and Runyan et al., do not use remote sensing data collected by UAVs, they not only significantly advance aspects of the algorithms that are used when processing UAV data, but also represent interesting applications that showcase the current practical impact of Deep Learning networks. Silva Aguiar et al. present a SLAM algorithm aimed at vineyard environments. The processing of the point data obtained with a LIDAR sensor mounted on a land robot, and in particular the combined use of a point-based and semi-plane based environment mapping technique has the potential to improve existing point cloud analysis techniques used for other sources of data such as, for example, the detection of individual trees or plants in forestry or agriculture applications. The authors of the paper in particular mention the possibility to extract features with semantic importance such as trunks and mention the need to integrate these semantic features in the automatic processing of the data. The future research directions outlined by the authors include some of the advances presented in the other papers in this Research Topic, showing how even long established research areas such as SLAM can benefit from the current developments in drone technology and in image analysis.

The final contribution, Runyan et al. shows the potential and the current limitations of Deep Learning—based image analysis techniques. The authors of Runyan et al. present a study where underwater image mosaics and point clouds were used to study the evolution of a coral reef environment over several years. Remarkably, the data processing pipeline https://www.overleaf.com/project/6329074e4f4f733ee854558b described in this paper is the same as that used in UAV image analysis down to the software used for mosaic and point cloud construction. Similarly, Deep Learning networks such as ResNet He et al. (2016) are used, as is common in many UAV-based applications. As a major point of interest in this contribution, several deep learning networks with different characteristics and using different dimension data (2D, 2.5D, and 3D) are used but the results obtained are mentioned as one of the limitations of the study.

We believe that the four contributions presented, when taken together, show the rapid pace of development of this researcher area, its many accomplishments and also picture some of its current limitations and evolution possibilities. We hope the high quality research presented in them is of interest for readers and helps sparks future research developments.
